# Reconciling differences pertaining to the origin of SARS-CoV-2

**DOI:** 10.1186/s42269-022-00712-4

**Published:** 2022-02-08

**Authors:** Yasin Ali Muhammad

**Affiliations:** Department of Biological Sciences, Winston-Salem State University, Winston-Salem, NC USA

**Keywords:** SARS-CoV-2, COVID-19, Virology

## Abstract

**Background:**

At the time of this writing, SARS-CoV-2 has reportedly claimed the lives of millions of people worldwide. However, there is still disagreement concerning the origin of SARS-CoV-2, its true nature, and the extent of its pathogenicity. Thus, the purpose of this manuscript is to highlight and critically analyze these differences so that research efforts can be geared toward addressing these concerns.

**Main Body:**

For this purpose, the author studied the perspectives of both conventional and non-conventional scientists, physicians, and researchers in an attempt to understand the points of contention and the reasons for the vast gulf in perspective.

**Conclusion:**

After reviewing the varying but divergent perspective pertaining to the origin of SARS-CoV-2 and the premises used to justify them, it has become clear that if the scientific community is to put a halt to the spread of misinformation pertaining to the origin of SARS-CoV-2 and COVID-19, the predominant scientific community (particularly the microbiologist/immunologist) must carry out the requisite scientific procedures and encourage governmental/academic transparency.

## Background

To date, there are three prevailing theories regarding the origin of what is now referred to as “SARS-COV-2.” The dominant consensus (dominant as in most popular) says that SARS-COV-2 is a novel and naturally occurring coronavirus that became infectious to humans through zoonotic transmission. In stark contrast from the former, the second theory argues that this SARS-CoV-2 had its genesis in a laboratory headed by researchers from the Wuhan Institute of Virology (WIV), under the auspices of Anthony Fauci and the National Institute of Allergy and Infectious Disease (NIAID) for “gain of function” research (Kennedy 2020; Yan et al. 2020; Breggin and Breggin 2020). And then there’s the latter, which is that there is no novel and highly contagious pathogen on the loose that has, essentially, been isolated, purified, and shown to cause disease in of itself in an experimental setting (Lanka 2020; Cowan and Morell 2020; Kaufman 2020). Considering all three positions are heralded by qualified researchers, scientists and physicians, why is there such a gulf in perspective? All three proposals will be discussed further in the latter portions of this text, but first, some background knowledge concerning the discovery of SARS-COV-2 is warranted.

On December 30, 2019, Dr. Li Wenliang reached out to several of his colleagues via “Wechat” (a China based communication app) to inform them that, at the hospital in which they worked, some of the patients were placed into quarantine after falling ill with a condition resembling Severe Acute Respiratory Syndrome. Shortly afterward, one of the individuals on the receiving end of the message decided to leak the information Dr. Wenliang presented them with, and of course, the word spread rapidly thereafter (Green 2020). According to the World Health Organization (WHO), on December 31, 2019, the WHO China office was apprised of similar cases of atypical pneumonia of unknown cause in Wuhan, China; later, on January 24, 2020, and February 3, 2020, respectively, scientist from the Chinese Centers for Disease Control (CCDC) published their initial reports demonstrating the acquisition and assimilation of relatively short sequences of genetic material that were eventually deemed to be representative of the genome of SARS-COV-2 (then called nCOV-19), along with electron micrographs of particles present during/after cell culture experiments. However, prior to the release of these publications, the WHO endorsed the use of an RT-PCR test, made by Professor Christian Drosten from the Institute of Virology at the Charite Medical School in Berlin, by countries all over the world (Corman et al. 2019). Strange is how the WHO and Professor Drosten could recommend and deploy the use of a test for a novel virus prior to the presence of a peer reviewed publication demonstrating its existence. Another question one might ask is how could such a test be judged as reliable and fit for diagnostic use on the physically ill? Note, Professor Drosten began producing an assay for this new virus on January 1, 2020, six days before the Chinese is said to have isolated the new virus (Ellis et al. 2020; World Health Organization 2020). Most readers of this paper will be familiar with, at least, the concept of PCR and how it works in theory; but even for those with this background knowledge, it is important to reexamine the nature of PCR tests in general and the RT-PCR test(s) for SARS-CoV-2 in particular.

**Fig. 1:**
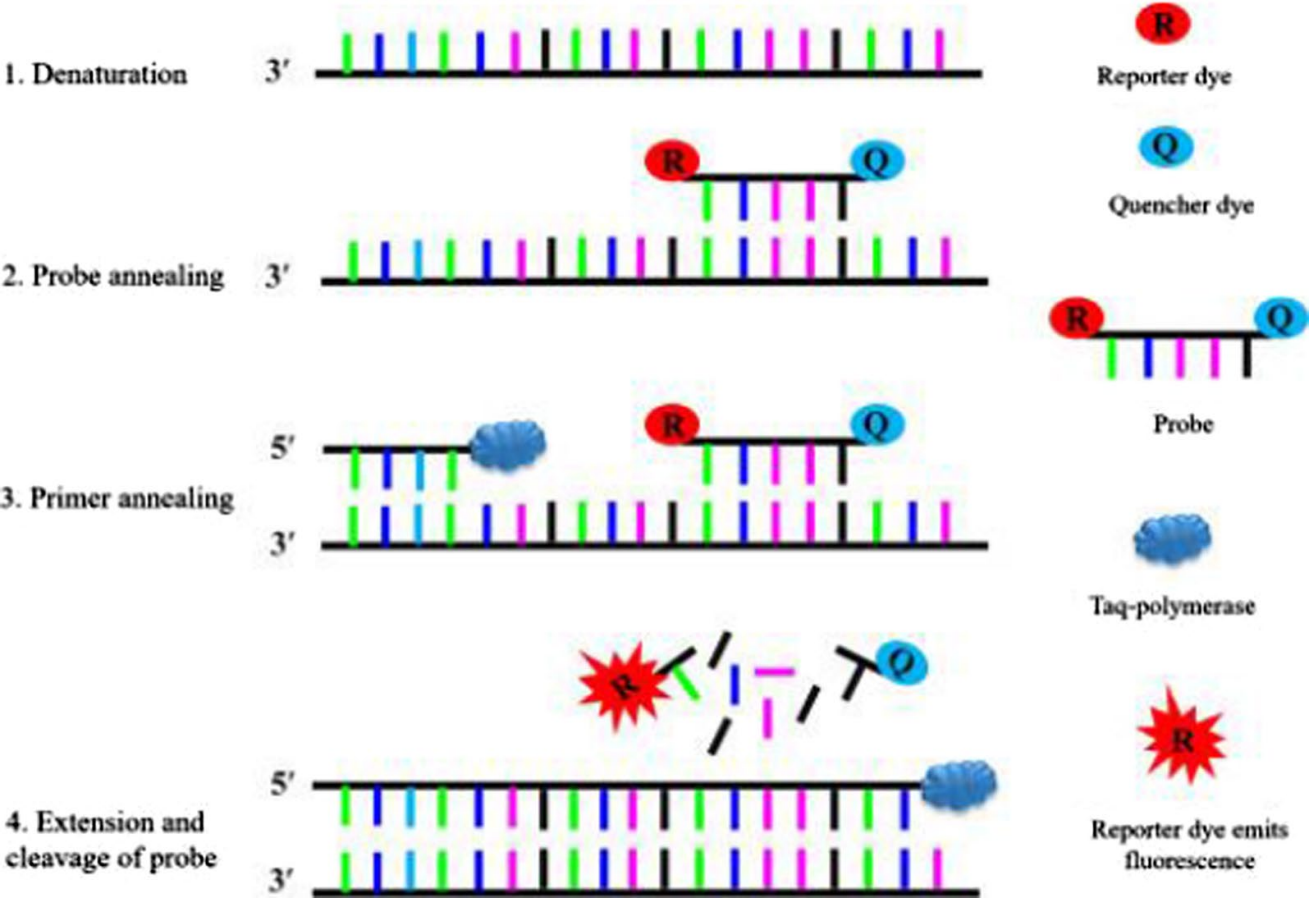
Reverse transcription polymerase chain reaction (RTPCR) (Roy et al. 2019)

**Fig. 2:**
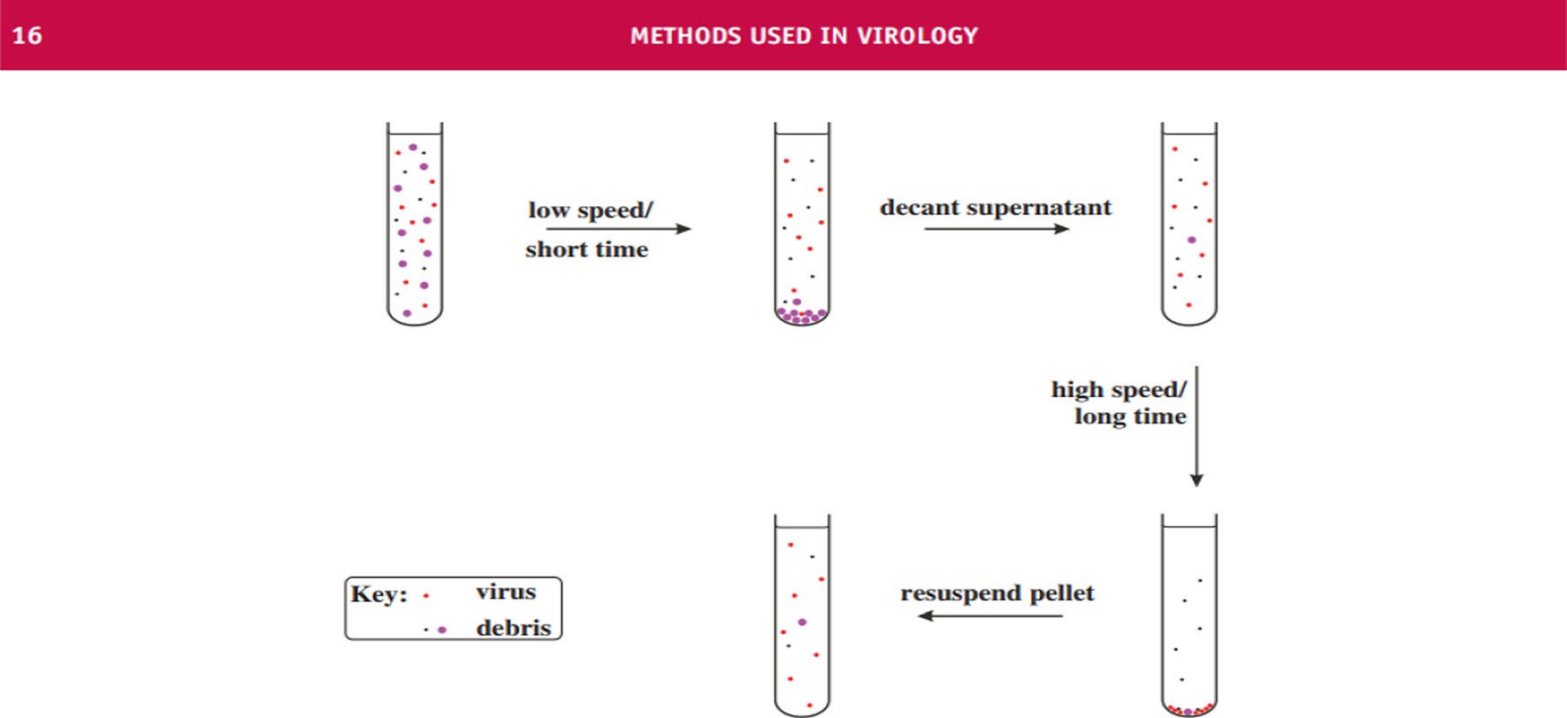
Partial purification of virions by differential centrifugation. A crude preparation of virus containing host debris is subjected to low-speed/short-time centrifugation (e.g. 10,000 g/20 min) followed by high-speed/long-time centrifugation (e.g. 100,000 g/2 h. This cycle can be repeated to obtain a higher degree of purity. The final pellet containing partly purified virus is re-suspended in a small volume of fluid (Carter and Saunders 2007)

**Fig. 3:**
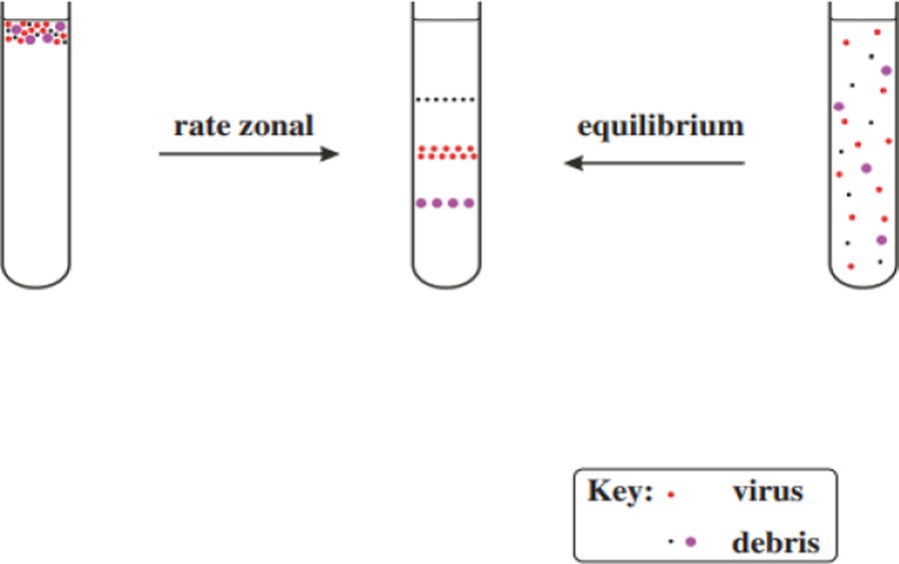
Purification of virions by density gradient centrifugation. A partly purified preparation of virus is further purified in a density gradient. Rate zonal centrifugation involves layering the preparation on top of a pre-formed gradient. Equilibrium centrifugation can often be done starting with a suspension of the impure virus in a solution of the gradient material; the gradient is formed during centrifugation (Carter and Saunders 2007)

Reverse Transcription Polymerase Chain Reaction (RT-PCR) is a technique designed to multiply or “amplify'' relatively short fragments of genetic material present in a sample from sequences that are otherwise difficult to find. Here, small (roughly a few dozen base pairs) pre-determined sequences (primers) are designed to complement target sequences in order to act as molecular tags for the identification of genetic material associated with particular kinds of viruses. This “matching” (annealing) process then sets off a cascade of events that facilitates the multiplication and identification of target sequences. Thus, one must use a template strand, from a database, that is a homolog of a hypothetical full genetic sequence (the one to which the new virus may possess) to generate and use these primers/probes. And under ideal circumstances, this template strand should have been removed from a prior virus that was isolated and compared to a newly isolated and purified virion in the same manner (more on this later). Below is an attachment summarizing the RT-PCR process and the purification process via Density Gradient Centrifugation (DGC), respectively (Figs. 1, 2, 3) (Roy et al. 2019; Carter and Saunders 2007).

On January 1, 2020, Dr. Christian Drosten downloaded a virological database describing the genomic structure ascribed to the original SARS virus (SARS-CoV). He then used this sequence as a foundation for which to make a test for what eventually became known as SARS-CoV-2. From the original template, he made primers that would be used as “tags” to be the standard bearer for whether or not these complimentary but hypothetical sequences were present in the sputum samples of sick patients. On January 23, 2020, Professor Drosten and his colleagues published a paper where he described the pivotal events that preceded his course of action, he says “Before public release of a virus sequence from cases of 2019nCOV, we relied on social media reports announcing detection of a SARS-like virus. We thus assumed that a SARS-related CoV is involved in the outbreak” (Ellis et al. 2020). Afterward, in an attempt to fully elucidate the full genome of this novel virus, Chinese virologists used computer programs to align and “fill genomic gaps” in order to assimilate their semi-discontinuous genetic sequence for nCoV19 into a full genetic strand (Zhu et al. 2019; Peng et al. 2020). The first official compilations of genetic sequences, as noted earlier, were published on January 24, 2020, and on February 3, 2020, respectively, thus fulfilling the expectations of the public and the predominant scientific community (Zhu et al. 2019; Peng et al. 2020).

## Main text

### Theory number one, a natural pathogen

According to the natural origin or zoonotic transmission model, nCOV-19 is the “seventh coronavirus known to infect humans” and “is not a laboratory construct or a purposefully manipulated virus” (Andersen et al. 2020). Researchers hypothesized that this newly reported virus had originated from a wet market in Wuhan, China, during the early stages of the pandemic. Eventually, that hypothesis was discarded as 14 out of the initial 41 cases linked to the wet market had no apparent connection to it, plus; none of the samples obtained from the wet market tested positive using the RT-PCR method. Consequently, the Chinese CDC concluded, “further research shows no connections between food sold in Wuhans Market and the coronavirus” (Market and not Origin of COVID-19,2021). Although no proponent of the zoonotic transmission theory claims to know the exact origin of SARS-COV-2, one of the most popular and recent propositions comes from Dr. Kristian Anderson, an infectious disease researcher at Scripps Research Institute located in La Jolla, California, which suggests that there are sequences that belong to the genome of this new virus that differ radically from those belonging to any prior known virus. He then implies that for SARS-COV-2 to be a genetically engineered virus, one would have to use a previously present backbone from different pathogens, splice out the original virulence genes of this pathogen, and integrate it into the genome of this new coronavirus. However, according to the genetic analysis carried out by him and his team, there is no evidence to support the notion that such a task has been carried out.

Additionally, they maintain the Receptor Binding Domain (RBD), which is a region of the spike protein that is thought to optimize viral adsorption to the cell, differs from the RBD sequence previously predicted, using computational analysis, to be optimal for SARS-CoV and other SARS-like coronaviruses, in other words; if readily available and efficient RBD sequences were not used for the spike protein of SARS-CoV-2, then such sequences could not be used as a template during the laboratory synthesis of a new SARS-like coronavirus. By extension, the possession of a polybasic (furin) cleavage site by SARS-CoV-2 at the junction of subunit one (S1) and subunit two (S2) of its spike protein, enables the cleavage of proteins by furin (found in humans) and is thought to enhance the infectivity of the virus, creates the possibility that the addition of a polybasic cleavage site occurred during human to human transmission via natural selection (Nao et al. 2017). Dr. Anderson does entertain the possibility of serial passage, whereby laboratory scientists pass bat SARS-CoV-like viruses through cell lines, thus facilitating the natural selection of RBD mutations. However, there is no documentation regarding the existence of previously identified coronaviruses that have acquired analogous or sufficiently homologous RBD’s to that of SARS-CoV-2. To further support the natural origin theory, RaTG13, a bat coronavirus sampled from the *Rhinolophus affinis* species, exhibits 96% sequence homology to SARS-CoV-2; however, its spike diverges in the RBD, suggesting that it may not bind with the efficacy SARS-CoV-2 is thought to possess (Wan et al. 2020). Despite this, he and his team are optimistic due to the presence of more homologous RBD residues (amino acid arrangements) in some coronaviruses found in an illegally imported species of animals called Malayan pangolins, thus alluding to the possibility of a genetic recombination event between bats, pangolins, and humans. However, as noted by Anderson et al., “No animal coronavirus has been identified that is sufficiently similar to have served as the progenitor of SARS-CoV-2”.

### Theory number two, a laboratory made virus

The funding of Chinese scientists, military virologists and their American counterparts for coronavirus and “gain of function” research is well-known and well documented. The purpose of this section is not to elucidate on the history of coronavirus research and “gain of function” research, but rather to assess the scientific plausibility of a laboratory origin for SARS-CoV-2.

In September of 2020, Li-Meng et al. published a report titled "Unusual Features of the SARS-CoV-2 Genome Suggesting Sophisticated Laboratory Modification Rather Than Natural Evolution and Delineation of Its Probable Synthetic Route," in which they proposed, as the title of the paper suggests, that SARS-CoV-2 is a laboratory construct (Yan et al. 2020). In general, their premises for presenting such an argument are based on genomic similarity between SARS-CoV-2 and ZC45, a "bat coronavirus discovered by military laboratories in the Third Military Medical University (Chongqing, China) and the Research Institute for Medicines of Nanjing Command (Nanjing, China)," similarities within the receptor-binding motif (specifically the S1 portion or first half of the spike protein) between SARS-Cov-2 and SARS-CoV, and the presence of the previously mentioned furin cleavage site present in the spike protein of the new virus, of which they contend, has never been predicted to be present for this particular genus of viruses (*Betacoronavirus*) until the appearance of SARS-CoV-2. Let us probe into each premise further.

The first premise is predicated on the existence of a coronavirus called ZC45, which shares high sequence homology with SARS-CoV-2 (on the nucleic acid and amino acid level). Specifically, on the amino acid level, the authors note that the nucleocapsid (NC) proteins are 94% identical, the membrane (M) proteins are 98.6% similar, the S2 portion or second half of their spike proteins are 95% similar, their open reading frame 8 (ORF8) proteins are 94.2% identical, and their envelope (E) proteins are 100% equivalent. This appears to be a fatal blow to the natural origin theory because while ZC45 and SARS-CoV-2 share 94.2% sequence identity on ORF8, no other coronavirus shares more than a 58% sequence identity with SARs-CoV-2 for ORF8; thus, they believe it reasonable to infer that the SARS-CoV-2 ORF8 protein was made from the backbone of ZC45. Additionally, as they mention, although 100% sequence identity on the E protein has been documented for SARS-CoV and other SARS-related bat coronaviruses, none of them at the same time share greater than 83% homology on ORF8 (Wan et al. 2020). Thus, the seemingly anomalous simultaneous genetic similarities, in their opinion, are consistent with the laboratory origin theory. Additionally, Dr. Yan and her colleagues are not the only team of Virologist who’ve reported on the close phylogenetic relationship between ZC45 and SARS-CoV-2, as the Shanghai Public Health Clinical Centre (a BSL-3 laboratory) also suggested a link between the two viruses based on their striking genetic similarities (Global Defense Staff 2020).

Similar to what Dr. Yan and her team describe for the previously mentioned viral proteins, the S2 portion of the SARS-CoV-2 spike protein shares high sequence identity (95%) with that of ZC45; however, the S1 part of the spike is only 69% homologous to that of SARS-CoV-2. Thus, the spike protein (particularly the S1 portion) of the two viruses differs significantly, though the S1 part (however, not an exact match) shares similarities to that of SARS-CoV. Actually, most of the amino acid residues (from SARS-CoV) essential for hACE2 binding and protein folding are present (Yan et al. 2020; Yang et al. 2015). Concerning the polybasic cleavage site, which refers to a specific position at the junction of the S1 and S2 portions of the spike protein, they maintain that this site is not present on the spike proteins of any of the other coronaviruses of the same genus (*Betacoronavirus*) thus expanding its cell tropism and therefore might have been inserted. To further support this argument, the amino acid arrangement comprising the polybasic cleavage site contains two consecutive Arginine residues in between proline and alanine, meaning that the codons used to translate these amino acids are CGGCGG (CCG is considered a rare codon) of which is only found at one particular location in the SARS-CoV-2 genome; thus, the fact that this “rare codon” is used in tandem in only one specific area seems to suggest that this arrangement could not have been a chance occurrence.

Interestingly, the Fau1 restriction site is formed by this particular arrangement of codons, which, they contend, is evidence that "restriction fragment length polymorphism," which is a technique that the Wuhan Institute of Virology specializes in, could have been used. Yan and colleagues also mention two other restriction sites on either side of the SARS-CoV-2 genome, namely EcoR1 and BstE2, which are commonly used restriction sites for molecular cloning. Lastly, it is worthy to note that Dr. Zhengli Shi, a Virologist from the Wuhan Institute of Virology, and her colleagues "swapped" a SARS-CoV RBM into the spike (S) genes of another bat coronavirus referred to as SARS-like coronavirus (SL-CoV); they did this, ultimately, by synthesizing the S gene of SARS-CoV, cloning the S gene of SL-CoV, and then replacing the target regions of SL-CoV with the desired counterparts of the S gene of SARS-CoV (Ren et al. 2008). And recently, an active collaborator of Dr. Shi, along with nine other researchers, "swapped" the genes for the RBM attributed to SARS-CoV-2 into that of SARS-CoV by synthesizing their spike genes and subcloning them into a pFastBac vector (Bac-to-Bac system) for the production of recombinant baculoviruses in insect cells (Shang et al. 2020). In both cases, the goal was for non-coronavirus vectors (either pseudoviruses or baculoviruses) to express coronavirus genes. Furthermore, Dr. Yan and colleagues note that similar restriction sites are also found in both of these experiments; thus, they believe these facts to be a "smoking gun proving" that SARS-CoV-2 is the product of genetic manipulation.

It should be noted that the conventional scientific consensus is that a bat coronavirus called RATG13 (mentioned earlier in the paper) shares the most significant sequence homology out of any other previously mentioned viruses and therefore is the likely progenitor virus. However, RATG13 is excluded from the authors' representation of Yan et al. because she and her team excluded a thorough analysis of this virus in the report themselves. Dr. Li Meng Yan and colleagues argue in their document that the origin of RATG13 is questionable and has since published a preprint elucidating on their assertion; however, this is beyond the scope of this paper.

### Theory number three, no evidence of a dangerous new pathogen

On August 20, 2020, independent researcher Andrew Johnson received a response to a Freedom of Information Act Request (FOIA) he submitted to Public Health England (PHE), in which he requested documents describing the isolation (purification) of SARS-CoV-2 virions directly from the bodily fluids of patients said to be infected with the virus. A representative for PHE responded by saying, "I can confirm that PHE does not hold the information you have specified" (Public Health England 2020). Another researcher, of whoms' name remains anonymous, reached out to the UK Government Office of Science (GO-Science) asking for a scientific reference describing the isolation (purification) process of SARS-CoV-2; similarly, GOScience responded, "We do not hold the information you have requested" (Government Office for Science 2020). GO-Science then pointed the researcher in the direction of PHE and the broader Department of Health and Social Care (DHSC), though, as noted previously, PHE stated they have no records meeting such criteria, and DHSC, upon request reported: "DHSC does not hold the information you have requested" (Freedom of Information Request Reference FOI-1247803,2020). On November 2, 2020, researchers requested the same documentation from the Centers for Disease Control; they also stated, "A search of our records failed to reveal any documents pertaining to your request" (Centers for Disease Control and Prevention 2020). Requests such as these were also sent to Canadian Public Health authorities and yielded the same results (Christine 2020). Thus, the absence of viral isolation or purification, as acknowledged by these prominent public health authorities, in the opinion of some, suggests that the proper methods by which contagion and viral pathogenicity is proven have not been deployed. In theory, per Koch's Postulates and Rivers criteria (named after the German bacteriologist Robert Koch and American Virologist Thomas Rivers, respectively), microbial or viral isolation is the first step one should take to properly assess the disease-causing capabilities of a microbe or a non-cellular entity. And according to those who are skeptical of the existence of, or even the dominant consensus concerning, SARS-CoV-2 or the extent of its disease causing capabilities, any test or experiment predicated on methods excluding this necessary but fundamental piece of evidence is rendered null and void.

Recall, diagnostic tests (RT-PCR) are designed to amplify (multiply) short sequences of genetic material (representing genes) that are associated with SARS-CoV-2, but first, these genetic sequences must be identified using complementary sequences (primers and probes). The question then becomes, how are specific molecular tags (primers and probes) for particular sequences from a new virus (in this case, SARS-CoV-2) made, considering a template strand directly from a purified virion has never been obtained. As noted by Professor Drosten, the first scientist to have developed a diagnostic test for what became known as SARS-CoV-2, he and his team "aimed to develop and deploy robust diagnostic methodology for use in public health laboratory settings without having virus material available … It's design relying on close genetic relatedness of 2019-nCoV with SARS-coronavirus, making use of synthetic nucleic acid technology" (Ellis et al. 2020). Put another way, the initial molecular tags used in the initial test for this new virus relied on pre-determined sequences, present in a genomic database, from the genome ascribed to SARS-CoV. Later primers were synthesized based on the foundation laid down by Professor Drosten and were subsequently ascribed to SARS-CoV-2 after de novo assembly of the new viral genome from non-full length strands of genetic material with the help of sequence alignment technology. According to one of the teams that reportedly elucidated on the genome of SARS-CoV-2, supernatant from patient bodily fluids (lung fluid and sputum from oral swabs) were obtained and prepared for RNA extraction (without pretreatment) or added to "viral transport medium" for the "isolation of the virus" (Peng et al. 2020). Following RNA extraction from viral culture supernatant and bronchoalveolar lavage fluid, Next Generation Sequencing (NGS) was used to assemble the genome of SARS-CoV-2, and further characterization was done by way of metagenomic analysis.

Thus, the assimilation of a viral genome and the production of tests (diagnostic and otherwise) for SARS-CoV-2 do not necessitate the presence of isolated (purified) virions. Disconcerting then is how there are many scientific papers in which authors claim to have isolated this new virus. What is the meaning of this? One should understand that the term "isolation" is a semantic one and therefore means different things to different people. As alluded to previously, the term isolation is quite self-explanatory; it refers to the separation of variables from one another and is often thought to be synonymous with purification. However, the study authors of the Coronavirus papers, when using the term, do not refer to the purification of virions directly from the lung fluid of patients but are referring to the acquisition, inoculation, and culturing of supernatant and the subsequent presence of Coronavirus appearing particles (virions) thereafter, or the presence of genetic material associated with coronaviruses (per genetic sequences in databases), or a positive PCR reaction. The question then becomes, what's wrong with the use of the previously mentioned procedures?

Of course, if the genetic sequences that are being targeted by the primers for the COVID-19 diagnostic tests are specific to SARS-CoV-2, if the presence of Coronavirus appearing particles are indeed clearly the result of exogenous viral transmission, and if there are no confounds present within the viral culture experiments, then no issue exists. However, critiques of the conventional consensus argue that such may not be the case.

As noted previously, the assimilation of the SARS-CoV-2 genome is predicated on the presence of sequences of genetic material within supernatant that shared homology with sequences ascribed to the Coronoviridae family of viruses, and most intensely with SARS-CoV. So even though SARS-CoV-2 has not been purified, and even though genetic material has not been removed directly from a purified SARS-CoV-2 virion, it would seem within reason to do de novo assembly for a new SARS-related coronavirus if the primary template strand was available. However, there is no record of the purification of SARS-CoV or any of the previously identified common-cold associated coronaviruses either (Yasin 2020). The original reference article describing the isolation and identification of the SARS-CoV virus relied on the presence of virion appearing particles present within "fetal rhesus kidney cells'' from the lung biopsy and nasopharyngeal aspirate of two patients (Peiris et al. 2003), of which were seen through an Electron Micrograph (EM) image following the inoculation of supernatant onto the cell lines. In addition to the presence of virion appearing particles consistent with coronavirus size and morphology after viral culture, genetic material was extracted or precipitated from the sample in which the particles were obviated. Then, to ascertain the genetic sequence of the unknown virus, a random RT-PCR assay, in which random primers were used to detect coronavirus-associated genetic material, was employed. After analyzing the results of this assay, the study authors deduced, "Phylogenetically, human pneumonia-associated Coronavirus was not closely related to any known human or animal coronavirus or torovirus. We based our analysis on a 646 bp fragment of the polymerase gene which showed that the virus belongs to antigenic group 2 of the coronaviruses, along with murine hepatitis virus and bovine coronavirus" (Peiris et al. 2003). Additionally, it should be noted that, according to the study authors, "sequence analysis of this DNA fragment suggested this sequence had a weak homology to viruses of the family of Coronaviridae," sharing highest homology (57%) to the RNA polymerase of bovine Coronavirus and murine hepatitis virus (Peiris et al. 2003). Thus, the use of the genome ascertained for the original SARS-virus as a gold-standard or a template by which to assemble a new SARS-related genome, to some, seems unreasonable since one can't be sure what the origin of the template sequence is based on (because of lack direct removal from purified virions).

Of course, it can be argued that the cytopathic effects observed in cell culture are evidence of a novel pathogen. However, the skeptics contend that in the absence of confounding factors, one cannot be sure the particles found at the site of cellular damage are indeed its cause or at least, solely its cause. Typically, when cell culture experiments are done (particularly those for SARS-CoV and SARS-CoV-2), chemical agents such as antibiotics (usually two or more, and maybe antimycotics) are added to supernatant from a patient before incubation; this is done to exclude the presence of live bacteria that could potentially act as a confound during the experiment. However, being that broad-spectrum antibiotics belonging to the aminoglycoside class of drugs (particularly streptomycin, amphotericin-B, etc.) can be noxious to a variety of cell types (such as kidney cells and, if exposed, airway epithelial cells), there is a possibility that the antibiotics themselves might confound the results. Study authors should therefore control for these factors by purifying the virions directly from the samples of patients (presumably where there is a high viral load, then exposing these purified virions to the cell line of choice), expose the same kind of tissue to the supernatant/antibiotic mix, and then maybe conduct a third experiment whereby impure supernatant mixed with antibiotics is incubated along with the cell lines to exclude the possibility that the virions are acting as vectors for the potential confounds. Researchers should also control for the presence of exosomes and other virus-like particles (VLP), as these are frequently present in cell cultures where cells have been exposed to antibiotics (Németh et al. 2017) or other noxious substances; and can quite often, look much like, if not analogous to, the viruses most life scientists regard as pathogenic, including SARS-CoV-2, HIV, and others (Cassol et al. 2020; Gonelli et al. 2019; O'Hara et al. 1988).

Additionally, they can be present in cell cultures incubated in supernatants from patients who presumably do not have the infectious virus present as deduced by a negative antibody test or RT-PCR test either as artifacts of the disease process or as artefacts of the cell culture experiment (Gonelli et al. 2019). Another reason to control for these particles is that not only are they often present in the supernatant; they also frequently band at the same density as viruses after DGC. Of course, one could contend that the distinguishing factor between these VLP’s and live virions is that patient samples and cell cultures harboring them do not test positive on diagnostic or serological assays. However, it should be noted that a negative test result (either from an RT-PCR tests or an immunohistochemical analysis) is not necessarily a reliable distinguishing factor, considering patient samples and tissues, for a variety of reasons, can yield variable results using both types of assays (Cassol et al. 2020; Young et al. 2020; Li et al. 2020). Even Peng et al., the authors of one of the seminal SARS-CoV-2 papers, mentioned that patients who initially tested positive using an RT-PCR assay, may test negative upon retesting. Also, people who aren’t ascertained to have any viral-related illness may often have viral-pathogen associated genetic material in their blood (Moustafa et al. 2017). Free genetic material should also be screened for considering it is commonly present in humans' lung fluid. By extension, the releases of genetic material from cells following cells' death during cell culture experiments (more or less due to potential confounds) may also interfere with genome sequencing endeavors.

Finally, worthy of note is that in vitro experiments are not the only ones done, animal studies are also usually conducted to ascertain infectious causation, though there seem to be confounds present within these experiments also. The first in vivo experiment done for SARS-CoV-2, published by Bao et al., exposed mice transgenic for the human ACE-2 receptor (hACE2) and wild-type mice to supernatant from cell culture incubated with human lung fluid via intranasal inoculation. Later, the study authors conducted autopsies and displayed the lungs of three different mice (one from a transgenic mouse, one from a wild type mouse, and one from a control mouse exposed to PBS). Herein, the lungs from the transgenic mouse and wild-type mouse both displayed pathological changes, but the lung from the transgenic mouse displayed evidence of moderate interstitial pneumonia. Thus, the study authors deduced that the presence of hACE2 in the transgenic mouse made it more susceptible to the disease-causing capabilities of SARS-CoV-2, presumably because it binds strongly to hACE2. However, one could raise the question, what happened to the other mice? Were the results variable or were they consistent throughout? Were autopsies done for all of the mice? The authors do not address these concerns. Additionally, most readers who hear the term “intranasal inoculation” are often under the impression that intranasal inoculation entails the rubbing of supernatant fluid or purified virions along the borders of the nasal cavity of the test subject. However, this is not the case; as such procedures can be quite aggressive and even a bit invasive. Mice are often inverted, given anesthesia and micropipettes are used to shoot fluid down their trachea into their lungs for anywhere from 30 s to a minute, held up by their ears where rapid breathing and inhalation is facilitated (maybe prior to inversion, to facilitate the rapid inhalation of the supernatant); or more invasive and novel methods can be used to inoculate mice, wherein the use of a microsyringe pump is used to deliver substances, and these procedures can often be quite irritating to the mice as evidenced by their tendency to aspirate the inoculum (Dilini 2015; Kanazawa et al. 2018; University of Alabama xxxx). However, the exact inoculation procedure used for the mice study was not specified. Additionally, in the case of the aforementioned experiment, mice are not exposed to purified virions but instead supernatant containing the collection of substances mentioned previously (such as broad spectrum antibiotics) of which are hardly innocuous and has been implicated in drug induced interstitial pneumonitis (Machida et al. 2013). Could the presence of such substances, plus the presence of particles (virions) with a high affinity for hACE2, act as a confound and exaggerate the capabilities of the virus, whereby the virions are behaving like vectors for the confounding factors? Such a suggestion is far from farfetched, being that viruses are solid objects that could easily interact with matter such as pharmaceutical agents and facilitate their distribution; plus, such phenomena have been described for other noxious substances (Comunian et al. 2020). Is such a phenomenon being exemplified in these experiments? Considering that viral challenge experiments have not yielded results consistent with COVID-19 related autopsies (at least for severe COVID-19, the autopsy reports describe the presence of profuse alveolar damage, necrosis of pneumocytes, hyaline membranes, interstitial edema, metaplasia, and blood clots in arterial walls), the only way to know is to fulfill Koch’s postulates (or Rivers criteria) for SARS-CoV-2 and include proper positive and negative controls in both in vitro and in vivo experiments) (Carsana et al. 2020; Wichmann et al. 2020).

## Conclusions

To summarize, the proponents of the natural origin theory defend the correctness of their contention on several grounds, including but not limited to: high overall sequence homology between SARS-CoV-2 and RaTG13, distinct genomic and proteomic similarities between SARS-CoV-2, and SARS-CoV, distinct similarities between the RBD of SARS-CoV-2 and the previously mentioned pangolin coronaviruses coupled with their (Pangolins) being imported into Wuhan not long before small clusters of ill people were ascertained. Additionally, coronavirus appearing particles in the cell-cultures used to incubate patient samples and supernatant yielding positive RT-PCR test results after the assays were formulated provided the natural origin proponents with the evidence they needed to determine the causative agent of these patients' pneumonia.

The proponents of the synthetic route theory believe in the correctness of their contention based on but not limited to: similarities in the receptor-binding domain (S1 portion) of SARS-CoV-2 to SARS-CoV, implying that the RBD of SARS-CoV was used as a backbone for the production of the RBD for SARS-CoV-2, the presence of a unique furin cleavage site which has not (using proteomic models) been predicted as being present for any other virus within the *Betacoronavirus* genus of viruses (according to their analysis), the shared homology in the critical proteins between SARS-CoV-2 and ZC45 (E, NC, S2 portion of RBM, ORF8 and M proteins), the presence of Fau1, EcoR1, and BstE2 restriction sites which are commonly used regions for molecular cloning, and rare tandem repeats (CGGCGG) present within the SARS-CoV-2 genome.

And lastly, those who question either the existence of a novel pathogenic coronavirus or the extent of its pathogenicity do so because Koch's Postulates (or Rivers criteria) has not been fulfilled for this virus; and thus regard these experimental protocols as common sense approaches to proving microbial or viral pathogenicity. Additionally, they see the origin stories of the previously mentioned groups of scientists and researchers as speculative, being that diagnostic assays were made without an unequivocally ascertained template strand of viral genetic material removed directly from purified virions, since there are seemingly undeniable confounding factors present within all SARS-CoV-2 experiments, since (due to a variety of reasons) tissues can produce particles that are often regarded as morphologically indistinguishable from what are recognized as being pathogenic viruses, and since test results (both immunohistochemical and nucleic acid-based) can be quite variable, going from positive to negative depending a variety of factors.

In critique of the synthetic route theory, researchers from Johns Hopkins Bloomberg School of Public Health (JHSPH) attack a number of premises used to justify this proposition (Johns Hopkins School of Public Health 2020). For example, contrary to the contention of Yan et al., JHSPH maintains that the presence of a furin cleavage site in *Betacoronaviruses* is not unheard of, as the presence of a furin cleavage site has been predicted for MERS-CoV (Millet and Whittaker 2014). JHSPH also maintains that sequence analogy between the same class of viruses is not unheard of either, as sequence analogy has been recorded between SARS-CoV and previously characterized coronaviruses, though none of the previously identified *Betacoronaviruses* share 100% sequence homology with SARS-CoV-2. Interestingly, Yan et al. referenced a study by Zhang et al., suggesting that based on an in silico analysis, the RBD of RaTG13 (assuming it is the natural origin) was unable to bind to the ACE-2 receptors of two orthologous (closely related to but different from *R. affinis*) horseshoe bats, suggesting that (*R. affinis*) could not be the natural host of RaTG13 (Zhang et al. 2020). However, JHSPH notes such studies have not been done on (*R. affinis*), the type of bat that RaTG13 is thought to come from, and therefore, conclusions cannot be drawn from the study mentioned above.

Additionally, they maintain that the RBD of SARS-CoV-2 binds optimally to the ACE2 receptor of human, pangolin, and *R. macrotis* and therefore generalizations should not be made based on the perplexing findings of the study referenced by Yan et al. Furthermore, there is the matter of restriction sites within the genome of SARS-CoV-2; however, restriction sites often occur naturally in genomes and therefore are not uncommon occurrences , even the previously mentioned “rare codons” appear in the genomes of other viruses predating the emergence of SARS-CoV-2 (Maxmen and Mallapaty 2021). Lastly, the authors of the JHSPH critique of the Yan report assert that Yan and colleagues are cherry-picking similarities between ZC45 and SARS-CoV-2 and are refusing to acknowledge the significance of the differences. For example, the fact that the S1 portion of the SARS-CoV-2 RBD bears low homology (69%) to that of ZC45. Or that there are reportedly 3000 interspersed nucleotide differences between SARS-CoV-2 and ZC45, and thus seems improbable that a viable, synthetic virus could be made from the template of a virus with such qualities.

Still, JHSPH failed to address some of the other circumstantial evidence alluding to possible laboratory origin. For example, the repeated (but rare) codons present in the nucleotide sequence for the SARS-CoV-2 spike is, according to Yan et al., hardly a coincidence, coupled with the fact that this codon sequence forms the Fau1 restriction site alludes to the possibility that a technique called "restriction fragment length polymorphism," a method that WIV researchers are known to use might have been employed (Zeng et al. 2016).

Moreover, to critics of the laboratory origin hypothesis, the appearance of low pathogenicity or the manifestation of atypical disease following laboratory experiments in mice after they have been genetically engineered to be increasingly susceptible to infection makes it even more unlikely that SARS-CoV-2 was optimized for its pathogenic capabilities (either in wild-type mice or hACE2 mice) during the course of gain-of-function research. Furthermore, despite the WIV apparently exhaustive catalog of bat-derived SARSr-CoVs (SARS-related Coronaviruses), none of these viruses relate closely to SARS-CoV-2 (rather, they relate more closely to SARS-CoV) and ZC45 is not among that list of viruses (Holmes et al. 2021).

It is essential to keep in mind that circumstantial evidence on any side is not enough to ascertain origin and pathogenicity. Some contend that the presence of HIV-associated genetic material in the genomic structure SARS-CoV-2 justifies the argument that parts of HIV-related sequences might have been used to make SARS-CoV-2 (Pradhan et al. 2020), however, there are HIV-related sequences present in humans (Horwitz et al. 1992), plus, it is not uncommon for HIV appearing particles to be present in the bodies of human beings, as noted earlier in this text. Thus, such an argument should not be used to infer bio-engineering, especially without gold-standard experiments and a larger body of evidence to support such a conclusion.

Furthermore, scientists should be cautious about launching ad hominem attacks against other scientists (maybe in an adjacent field of study) and researchers because there have been quite a few documented instances in history when scientists have been wrong. One such example can be seen in Subacute Myelo-Optic Neuropathy (SMON), wherein Virologists, for 15–25 years, believed that SMON was caused by a particular virus when it was indeed iatrogenically induced (Duesberg 1996).

Ultimately, developing a better understanding of the origin of SARS-CoV-2 and the ways in which it has been studied, as well scrutinizing the experimental procedures used to ascertain its capabilities, will serve to provide the scientific and medical community with a better knowledge of how to develop treatments and prophylactics for COVID-19. Evidently, the structural characterization of a disease-causing virus is necessary for the purpose of developing therapeutics and prophylactics that target virus specific antigens. Due to the fact that purification ensures the complete or near-complete separation of virions from presumably non-viral material, many consider viral purification a necessity prior to the characterization of viral genetic material and proteins, especially in the event that the virus one is seeking to understand remains unknown (Killington et al. 1996; Bhat and Rao 2020).

Worthy of note is that affording scientists and researchers from adjacent or different fields of study, the opportunity to provide their input and test their hypothesis is what solved the SMON crisis. Hopefully, the scientific community at large will learn from its prior mistakes for the sake of the advancement of biomedical research.

## Data Availability

Visual materials were adapted from other texts.

## Abbreviations

CCDC: Chinese center for disease control
CDC: Center for disease control
COVID-19: Coronavirus disease 2019
DGC: Density gradient centrifugation
DHSC: Department of Health and Social Care
PHE: Public Health England
GO-Science: Government Office for Science
HIV: Human immunodeficiency virus
SARS-CoV-2: SARS Coronavirus 2
SMON: Subacute myelo-optico-neuropathy
RBD: Receptor binding domain
RBM: Receptor binding motif
RNA: Ribonucleic acid
RT-PCR: Reverse transcription polymerase chain reaction
WIV: Wuhan Institute of Virology
NIAID: National Institute of Allergy and Infectious Disease
JHSPH: Johns Hopkins School of Public Health
hACE2: Human angiotensin converting enzyme 2
ACE2: Angiotensin converting enzyme 2
FOIA: Freedom of information act
SL-CoV: SARS-Like coronavirus
NC: Nucleocapsid
M: Membrane
ORF8: Open reading frame 8
E: Envelop
SARSr-CoVs: SARS-related coronaviruses
